# Patients' perspectives on planned interventions tested in the Otago MASTER feasibility trial: an implementation-based process evaluation study

**DOI:** 10.1016/j.bjpt.2024.101086

**Published:** 2024-06-14

**Authors:** Daniel Cury Ribeiro, Amanda Wilkinson, Vander Gava, Sarah E. Lamb, J. Haxby Abbott

**Affiliations:** a Centre for Health, Activity and Rehabilitation Research, School of Physiotherapy, University of Otago, Dunedin, New Zealand; b Department of Nursing, University of Otago, Christchurch, New Zealand; c Department of Physical Therapy, Universidade Federal de São Carlos, São Carlos, Brazil; d University of Exeter Medical School, St Luke's Campus, Heavitree Road, Exeter, EX1 2LU, UK; e Department of Surgical Sciences, University of Otago Medical School, Dunedin, New Zealand

**Keywords:** Clinical trial, Exercise therapy, Pain, Process evaluation, Shoulder

## Abstract

**Background:**

Patients are key stakeholders of clinical research, and their perspectives are relevant for researchers when planning and conducting clinical trials. Numerous aspects of trial process can influence participants’ experiences. Their experiences within a trial can impact retention rates. Poor treatment adherence may bias treatment effect estimates. One way to improve recruitment and adherence is to design trials that are aligned with patients’ needs and preferences. This study reports a process evaluation of the Otago MASTER feasibility trial.

**Objectives:**

Our aims were to investigate the patients’ perceptions of the trial interventions through individual interviews.

**Methods:**

Twenty-five participants were recruited for the feasibility trial and were allocated to two groups: tailored or standardised exercise. Sixteen participants agreed to take part in individual semi-structured interviews. Interviews were transcribed verbatim, and all interviews were analysed thematically using an iterative approach.

**Results:**

Our key findings suggest participants: (1) took part in the study to access healthcare services and contribute to research; (2) valued interventions received; (3) reported certain barriers and facilitators to participate in the trial; and (4) highlighted areas for improvement when designing the full trial.

**Conclusion:**

Participants volunteered to access healthcare and to contribute to research. Participants valued the personalised care, perceived that their engagement within the trial improved their self-management and self-efficacy behaviour, valued the time spent with clinicians, and the empathetic environment and education received. Facilitators and barriers will require careful consideration in the future as the barriers may impact reliability and validity of future trial results.

## Highlights

• We identified key barriers and facilitators for patients to participate in the trial.
• Participants took part in the study to access healthcare services and contribute to research.
• Participants valued interventions received.
• Participants highlighted areas for improvement when designing the full trial.

## Introduction

Shoulder pain is a common musculoskeletal disorder.^1^ Within shoulder complaints, rotator cuff-related shoulder pain is the most common disorder and has slow recovery.^2^^,^^3^ Rotator cuff-related shoulder pain is defined as pain at the top and lateral part of the shoulder joint, that may spread to the neck and elbow, and is worsened by overhead activity.^4^ It is estimated that only 50% of patients presenting with new episode of rotator cuff-related shoulder pain fully recover within six months.^5^ Pending on treatment received (e.g., usual care or exercise therapy), recovery may take up to 12 months.^3^

Process evaluation studies help us to understand findings from clinical trials.^6^^,^^7^ The key components of process evaluation are contextual, implementation, and mechanism of impact.^8^ Contextual factors can inform theories of how the intervention works; and can affect or be affected by the implementation of interventions, their mechanisms, and outcomes. The implementation processes refer to how and what elements (e.g., fidelity, dose, adaptations to planned interventions, and reach) of an intervention is delivered.^8^^,^^9^ The mechanisms of impact refer to understand the causal pathways through which an intervention achieves its outcomes.^8^, ^9^, ^10^

Process evaluation studies should be conducted, ideally, at all stages of trials (from pilot or feasibility to implementation trials).^8^ When conducted alongside pilot or feasibility trials, these studies can inform researchers how to improve the design of the full trial.^8^, ^9^, ^10^, ^11^ When conducted alongside the full trial or during implementation trials, process evaluation studies help stakeholders to understand whether an intervention tested was (in)effective due to its implementation during the trial or its design.^8^^,^^9^

Patients’ experiences within a trial can affect retention rates and treatment adherence.^12^ By understanding participants’ experiences during a feasibility trial, researchers can amend and improve the design of the full trial. Numerous aspects of a trial can influence participants’ experiences, including administrative-related factors (e.g., information sheet, consent forms, questionnaires), design-related factors (e.g., time required, burden).^13^ Their experiences within a trial can impact on retention rates as well as on treatment adherence.^14^ Poor treatment adherence may bias treatment effect estimates^15^^,^^16^ and one way to improve recruitment and adherence is to design trials that are aligned with patients’ needs and preferences.^12^, ^13^, ^14^, ^15^, ^16^, ^17^ Given patients are one of the key stakeholders of clinical trials, their perspectives are relevant for researchers when planning and conducting clinical trials.^18^^,^^19^

In this paper, we expand the analyses reported in the MAnagement of Subacromial disorders of The shouldER (MASTER) feasibility trial.^20^ During the Otago MASTER trial, we recruited 28 participants who were randomly allocated into one of the following groups: (a) tailored exercise or (b) manual therapy and standardised exercise interventions. A total of 25 participants received interventions and completed all follow-ups. Interventions for both groups consisted of two sessions per week over eight weeks (i.e., 16 sessions in total). Each session lasted for approximately 45 min. We assessed the outcome measures at baseline and at 4, 8, and 12 weeks. One important characteristic of interventions planned for the MASTER trial was the high dose of interventions which does not reflect clinical practice in New Zealand.^20^^,^^21^ We have previously reported clinicians’ perceptions and treatment fidelity using a mixed-method process evaluation during the Otago MASTER feasibility trial.^11^ In this study, our aim was to explore participants’ perceptions of the trial interventions tested within the Otago MASTER feasibility trial.

## Methods

### Design

Using a qualitative descriptive approach,^22^^,^^23^ we explored participant views about participating in the Otago MASTER feasibility trial (registration number ANZCTR: 12617001405303). We were particularly interested in finding out about the participants’ perceived value of participating in the trial, along with any perceived barriers. Ethics approval for the feasibility trial was granted by the University of Otago Ethics Committee (Ref: H17/080). The original study undertook process of Māori consultation. We report this study following the Consolidated Criteria for Reporting Qualitative Research (COREQ).^24^ Patients or members of the public were not involved in the design or conduct of this study. The feasibility trial was conducted in Dunedin (New Zealand) and recruited participants aged from 18 to 65 years old with shoulder subacromial pain.

### Participants’ perceptions on planned interventions

Participants (*n* = 25) who completed the feasibility trial (received all interventions and completed all follow-ups) were invited to participate in an individual semi-structured interview. Participants were invited by the trial coordinator through e-mail and confirmed their interest (or not) by responding to that invitation by e-mail. Interested participants were provided with study information and a consent form. All participants provided signed consent to be interviewed. We conducted the interviews between April 2018 and April 2019. Recruitment continued until the research team agreed that no new information was added upon subsequent interviews suggesting data saturation had been reached.^25^^,^^26^ The semi-structured interview guide was developed by members of the research team with experience in qualitative studies and clinical experience in treating patients with shoulder pain (Supplementary material).

Interviews were held at a time and place of the participants choosing and were undertaken by three different members of the research team (DCR, the trial coordinator, and a research officer). DCR conducted two interviews, the trial coordinator conducted three interviews, and the remaining interviews were conducted by the research officer. DCR, male, is the principal investigator with clinical experience on shoulder rehabilitation and clinical research and has three years of qualitative research experience. The trial coordinator is a female physical therapist researcher, who did not know the participants and was trained by the research team to conduct interviews. The trial coordinator was trained prior to conducting the qualitative interviews. The research officer (DJ) has 15 years of experience with clinical and qualitative research, did not know the participants. Interviews were held in a quiet room within the School of Physiotherapy building.

None of the participants who agreed to take part in this study had a partner present during the interview. Before commencement of the interview, verbal consent to be digitally recorded was obtained from participants. Interviews were recorded using a digital voice recorder and lasted between 45 and 60 min. Interviews were transcribed by a private company. Transcripts were checked by the team but not returned to participants for verification.

### Data analyses and interpretation

We analysed data thematically using an iterative and inductive approach.^27^ To ensure anonymity, we allocated a number to each participant who was interviewed (from 1 to 16). Data were analyzed by three members of the research team (AW, DCR, and VG). AW has extensive experience in qualitative analysis and worked together with two other team members (DCR and VG) to analyse the data. VG is a physical therapist and graduate research student. All analysts were involved in all steps of data analyses. We familiarised ourselves with the data through multiple readings of the transcripts and recorded ideas linked to the research question, participants’ perceived value of and barriers to participating in the trial. Through multiple discussions data were coded, codes were tabulated along with representative participant quotes, and these were sorted into potential themes and a thematic map. Themes were defined through a consensus exercise and named and are reported below.

During data analyses, we explored whether participants had different perceptions and experiences according to the group they were allocated to. Through the data analyses, we identified themes and subthemes were similar for both groups. For that reason, we report themes and subthemes without referring to group allocation.

## Results

We invited all 25 participants who took part in the feasibility trial. Nine participants informed us they were not interested in participating and sixteen participants agreed to take part in the interviews. The demographic information for those who participated in this study are presented in Table 1.

**Table 1:** Demographics of participants.

	Participants (*n* = 16)
Age, year	47 (30 – 62)
Group	5 Standard / 7 Tailored
Weight, kg	87.8 (49.1 – 105.7)
Height, cm	176 (153 – 188.5)
BMI, kg/m²	28.3 (20.1 – 38.1)
Pain Duration, months	24 (1.5 – 384)
Occupation	Academic (*n* = 1) Chief Executive Officer (*n* = 1) Engineer (*n* = 1) Information technology (*n* = 1) Laboratory technician (*n* = 1) Manager (*n* = 2) Midwife (*n* = 1) Postgraduate student (*n* = 1) Registered nurse (*n* = 1) Residential designer (*n* = 1) Retail Assistant Cleaner (*n* = 1) Retired (*n* = 1) Undergraduate student (*n* = 2)

Data are median (range) or frequency. Kg: kilogram; cm: centimetres; m: metres.

We identified three main themes: (1) motivations to volunteer; (2) perceived value, and (3) barriers and facilitators.

### Motivations to volunteer

Participants took part in the study for personal reasons and to contribute to research (Participant 1 and 3, Table 2). Some participants took part in the study to find a solution to their shoulder pain (Participant 7, Table 2), after experiencing long-lasting pain that impacted on their ability to undertake daily living activities (Participant 2 and 7, Table 2), or to receive free treatment (Participant 4, Table 2). Other participants were seeking some form of diagnosis or reliable information concerning their condition (Participant 13, Table 2). Other participants had a desire to contribute to the greater good and reported a desire to contribute to the university research being undertaken and to benefit future patients who experience shoulder pain (Participant 1, Table 2).

**Table 2:** Motivations to volunteer in the trial.

Subtheme	Quotes
Personal reasons	I hoped that [participating in the trial would] reduce the pain, then like makes me able to do some stuff that I couldn't do [for the] last 10 years. (Participant 7) [I took part in the study] to see what you guys would do to help with the shoulder pain and the movement, and to see if you could diagnose my problem and maybe help come up with a solution of what I could do to help it. (Participant 2) I think my expectations were from a selfish point of view. The free physio would actually help me rehabilitate my shoulder. (Participant 4)
Diagnosis for shoulder	I'd had quite severe shoulder pain for a few months and so [the study] was something that I thought would be quite helpful to manage my pain and to maybe have some investigations into what was happening with my shoulder. (Participant 13)
Contribute to research	I've participated in lots of other studies and I find it's great 'cause I think it's really good to help people with their studies, but also to see the outcome of what happens with the study as well. So, I think it's beneficial for both parties. (Participant 1) I'd had shoulder pain for a couple of years and never did anything about it because it wasn't severe enough to stop me from living a normal life. I'd always thought “oh, it's something, I just need to go see a physio [about]”, but never did. So I just thought “oh I can contribute to a study and get my shoulder fixed at the same time. (Participant 3)

### Perceived value

The second theme, ‘Perceived value’, showed how participants valued the personalised care they received (Participant 1 and 14, Table 3). They were happy to commit to the time required of them (Participants 12 and 13, Table 3) and appreciated the empathetic environment (Participant 15, Table 3) and the information they received (Participants 3 and 7, Table 3). Participants valued the frequent supervised sessions with clinicians, as those sessions: (1) reinforced the need for and importance of home-based exercises (Participant 16, Table 3); (2) increased participants confidence with doing the exercises and managing their condition (Participants 6 and 15, Table 3); (3) motivated them to increase the exercise load (Participants 2 and 3, Table 3); and (4) provided an opportunity to learn about their condition. One participant described the relevance of supervised exercises, with progressions being better than their previous experiences with physical therapy treatments, in which, they received a fixed, non-changeable exercise programme (Participant 2, Table 3). Participants valued the explanations regarding the mechanisms that may have contributed to their shoulder condition (Participant 3 and 8, Table 3). Despite that, some participants still felt a need for having a specific diagnosis (Participant 2, Table 3).

**Table 3:** Perceived value from being part of the trial.

Subtheme	Quotes
Personalized care	I've found it's really good having the one on one with the physio, twice a week. I think that um doing exercises at home is great but having that commitment to come twice a week to a gym, being supervised, makes you do [the home-based] exercises I think that was a great benefit of it. (Participant 1) [It was] helpful to have instant feedback. And the adjustment [to exercises] while you were doing the exercises, that was really helpful… (Participant 14)
Frequency of sessions	Whereas my previous physio had shown me a couple of exercises, [then it was] “see you later”, [and] go home again [with no follow up]. (Participant 2) [The frequency] was right, yeah. I guess you needed the twice a week in a way. I don't know if it you could go down to once a week. (Participant 13)
Empathetic environment	If people enjoy participating, and that's reflected in the relationship between the staff and the participant. I think that's all you can ask for, really. (Participant 15)
Information	I was quite amazed actually how quick [I learnt], and I learnt quite a lot about the causes of [pain] while we were doing [the exercises]. (Participant 3) I can say the key strength was informing the patient. […] I ask question from my physiotherapist or she felt that she should describe why I'm doing that or why I don't do that. But it was the key strength [and] it was informative. (Participant 7)
Importance of home-based exercises	Yes. Like the exercises at home is more sort of maintenance and then getting [the exercises] extended here [in the clinic]. (Participant 16)
Confidence	I've now got this set of exercises that I'm used to that I can just carry on with at home. (Participant 6) [I've] certainly got a programme that you can continue on with… knowing that you got a little toolbox of your own that, maybe, you can use to help [your shoulder pain] going forward. (Participant 15) Yes. Like the exercises at home is more sort of maintenance and then getting [the exercises] extended here [in the clinic]. (Participant 16)
Motivation and load	I liked the plan, coming in and doing a routine. That was really good. And the progression, it kind of gave an aim [to achieve] something at the end of the eight weeks. So, [it] makes you want to work harder. (Participant 2) We kept stepping [the intensity] up and so I was generally always challenged and that was good 'cause I sort of needed that. (Participant 3)
Explanation about mechanisms	[The physiotherapist] was explaining how my shoulder had, well my scapula muscle, whatever you call it, wasn't engaging really anymore and that was overloading the littler muscles, causing the problem. So that self-awareness part really was an eye opener for me. (Participant 3) Turns out that [injury] had healed and working with a physiotherapist, it's actually motor control, is actually the muscles, the way I've adapted my muscles to hold the bones in place is totally wrong. […] I now know what the problem is. Whereas I didn't before. The ball of the joint's sitting in the wrong place. And I'm aware now that as soon as I feel that pain, stop, reset my shoulder. (Participant 8) It's about moving, it's about mobility of your shoulder, right? And that's what I've learnt, at least from this, is you don't want to just rigidly keep it in one place, and then do everything. You want to be mobile; you want to able to move your whole shoulder. (Participant 10)
Specific diagnosis	[I expected] maybe a diagnosis. Or [for someone] to say, "Yes this part's not working, you need to work on that." I think like pinpointing exactly what was going on in relation to the initial kind of injury, I'd like to know what I did to start off with. (Participant 2) Whereas with this intervention, I don't think anybody had tried to diagnose the problem, they were just going through a wide range of exercise that probably will generally help. (Participant 9)

### Barriers and facilitators

#### Logistics

The planned interventions required participants to attend two sessions per week over 8 weeks and this required them to plan their days to attend sessions. Participants valued the possibility to schedule all sessions in advance and, if required, the possibility to reschedule sessions when needed. They considered the scheduling of sessions went smoothly and were pleased with how easy it was to book appointments throughout the trial (Participant 12, Table 4). Some participants who did not live or work near the clinic mentioned the distance they needed to travel as being a barrier to participate in the study, with commuting time and parking being the key barriers (Participants 4 and 6, Table 4). Parking was offered to participants, and one acknowledged this was helpful (Participant 1, Table 4).

**Table 4:** Barriers and Facilitators to take part in the trial.

Subtheme	Quotes
Logistics – Scheduling sessions	Yeh, that [scheduling a session] was really easy. It was really good to have a list of all the appointments beforehand so I could just fill my diary and yeh that was definitely good. (Participant 12)
Logistics – Commuting and parking	I had to leave sort of 35–40 min to get from [home] through town, and depending on the traffic and road maintenance and stuff so, that was the only thing. (Participant 4) I live on the other side of town so that's the only factor for me but I'd have to come in and find a park. (Participant 6) I found that everyone was really helpful, you know likes of the receptionist getting me parking. (Participant 1)
Health Literacy	I got a little bit confused about the table to reduce the pain at the home exercise. (Participant 1) It wasn't that organised I think… some of the descriptions wasn't that clear for a non-specialist person like me. (Participant 7)
	[*Filling the booklet*] keeps you focused on what you're doing and why you're doing it and so that in itself was a motivator. (Participant 2)
	The scales were really confusing. You had to keep going, they weren't simple scales. I had to keep going back and forth, referring to what each number meant, that was, sort of crossover. […] I think I would've been better just to do, instead of filling it daily, fill it out weekly. Yeah, and quicker. And, also, just noticing the change over a week. I think that would be more significant, really. (Participant 14) I found the change of the pain scale, the change of the scales in the book. It's like oh, okay hang on I've gotta read this. Which way am I going this time, what's happening? (Participant 8) [The research assistant] was very helpful with that, especially with the disability. By the third time [I was completing it the questionnaire], I was surprised how much of it I could remember how I knew when the scales were changing from one end to another. (Participant 6)
Use of pictures and rationale for exercises	I think, generally, for people, I would say there needs to be more broader description. And just a sense of, maybe, where, what muscles they should feel, should be working at that particular time. (Participant 14) … showing me something better than a couple of pictures I think is required, especially if we can identify out of the protocol which ones are more likely to have a positive effect. So that I may want to continue doing them without supervision later on. I want to make sure I'm gonna get them right. I need to have something to refer to. (Participant 9) I found [the pictures] were quite good. (Participant 1) I couldn't say I have difficulty with [*the exercises and pictures*] … but if you want to publish it for general people, common people like me, I think [*there should be*] more [*pictures*]. [*The pictures were*] like an arrow showing the direction. (Participant 7)
Exercise barriers	No real barriers. But more for the home exercises, was more finding that time [to perform prescribed exercises]. (Participant 2) Sitting there counting to 10 multiple times and then waiting 30 s doesn't seem very intellectually stimulating, especially for people that aren't used to doing exercise. (Participant 9) A lot of the things that were done, I don't think, I don't know whether they had any benefit. Some of the exercises for example, it was like well perhaps they had no benefit at all. I'd like to know which ones are the ones that worked, ‘cause then I'll keep doing them. (Participant 9)
Clinicians’ perceptions and attitudes	I think the um practitioners I was with did less speculation about how I'm getting on because I'm following the protocol of this study regardless of what they think. […] And we know that they think a handful of the exercises are pointless for whatever [reason]… ‘oh we wouldn't do that, we wouldn't ask anybody to do this exercise but we're doing it anyway’. […] Although at one level, I wonder whether you could happily do that intervention using students as the facilitators rather than using teachers. (Participant 9)

#### Health literacy

The use of a logbook was perceived differently by participants. Some participants had problems filling the logbook due to clarity of instructions (Participant 5, Table 4) and considered the descriptions of exercises were unclear for a non-specialist audience (Participant 7, Table 4). On the other hand, some participants considered completing the logbook was not a problem and encouraged them to continue with the exercise programme (Participant 2, Table 4).

The scales of different questionnaires combined into one document was confusing for participants (Participant 8 and 14, Table 4). The scoring system was different between questionnaires, with some having low scores as better outcomes and other questionnaires having low scores as worse outcomes (Participants 8, Table 4). On the other hand, some participants adapted well to the types of questionnaires used and got used to the change in score direction, acknowledging the support from the research assistant to help them to complete the surveys (Participant 2, Table 4).

There were mixed perceptions about how useful the pictures included in the booklet were and some participants thought the exercise descriptions could also be improved (Participant 14, Table 4). Some participants thought more pictures were required to improve clarity of the instructions provided (Participant 9, Table 4), while other participants thought the pictures used were sufficient (Participant 1, Table 4). One participant did not have problems with the pictures used, but recognized these needed to improve if the aim was to share the exercises with other members of the community who were not health professionals (Participant 7, Table 4).

#### Exercise barriers

Participants noted that doing home exercises required motivation or time (Participant 2, Table 4). One participant thought the exercises were not interesting and was uncomfortable with the need to count the number of times the exercise was done (Participant 9, Table 4). Participants expressed interest in knowing the value and importance of prescribed exercises and were keen to be informed which one was beneficial for their condition (Participant 9, Table 4).

#### Clinicians’ perceptions and attitudes

Some barriers reported by participants reflected those perceived by the clinicians who delivered the interventions. Our findings showed that clinicians perceptions on the exercises included within the trial were transmitted to patients, who shared similar thoughts to those from clinicians (Participant 9, Table 4).

### Areas for improvement

#### Further information needed

Participants suggested more information regarding management of shoulder pain after completing their participation in the trial (Participant 1 and 9, Table 5), including the dose of home exercises to self-manage and general information (Participant 9, Table 5). They were interested in understanding which exercises were more effective for their condition, so that they could continue doing those exercises to manage their shoulder pain if needed.

**Table 5:** Areas for improvement.

Subtheme	Quotes
Further information needed	I've always found the encouragement in everything, and the best benefit is getting the results at the end and finding out what's actually happened and how people benefitted or didn't, or whatever. (Participant 1) Yes, [would appreciate a pamphlet or information sheet]. The more you inform people, not over inform them, they're less likely to go to something like Mr. Google and have a look, […] because then they see a lot of stuff that they could potentially get and to me that's over informed. […] You don't want [a pamphlet] to be too complicated, you just want it to be basic that people can just glance and read easily. (Participant 1) I'm looking for which is how can I find out what's the minimum set of exercise that will keep my shoulder in good condition. Which ones weren't helping and which ones were? I'd love to know. (Participant 9)

## Discussion

This qualitative study explored the perspectives of participants in a trial comparing a tailored intervention to a standardized strengthening programme. Our key findings suggest that participants: (1) took part in the study to access healthcare services and contribute to research; (2) valued interventions received; (3) reported certain barriers and facilitators to participate in the trial; and (4) highlighted areas for improvement when designing the full trial. As trialists, we need to be cognisant of the facilitators for participants and of the barriers, to enhance and mitigate these respectively, when planning the full trial.

Our findings suggest participants were motivated to take part in the trial due to access to healthcare (a service that they would not receive otherwise) and for a desire to give something back to the university or to research in general. These motivations are like those reported by patients with cardiovascular disease,^28^ who were interested in seeing the same clinician over time and keen to promote science. Contrary to our findings, those patients with cardiovascular disease rated access to free healthcare as least important and this may be due to the context of the healthcare system these participants came from. In New Zealand, patients who have chronic musculoskeletal pain (e.g., shoulder pain) have limited access to physical therapy services through the national healthcare system. In this study, we recruited participants from the community who were less likely to have easy access to appropriate healthcare.

Participants valued supervised sessions with a clinician and some participants reported those sessions increased their confidence and motivation to adhere to interventions (including home exercises). Participants reported developing self-management strategies and an increase in their self-efficacy behaviour. Self-efficacy is considered an important determinant of pain behaviour^29^ and was reported to be associated with better clinical outcomes in patients with shoulder pain.^30^ This study highlights the possible benefits of both interventions tested within the feasibility trial and seem to refute the idea that frequent sessions with a clinician would reduce self-efficacy, as participants reported feeling more confident to engage with exercises at home. Participants valued the empathetic environment. Through an empathetic relationship, clinicians can improve diagnostic accuracy, patient satisfaction, and their commitment to intervention requirements.^31^, ^32^, ^33^, ^34^

Participants expressed interest and value in being informed about the mechanisms that caused their shoulder pain. Interestingly, these were not part of the planned intervention within the feasibility trial and some of those focused on neuromechanical causes for shoulder pain. Currently, it is unclear what causes shoulder pain and it is very challenging to identify the potential source of symptoms in this population.^35^^,^^36^ Clinicians clearly shared their clinical beliefs with participants and these influenced participants’ perceptions of the prescribed exercises. Some participants also expected a diagnosis and more detailed explanation regarding the cause of their pain, which is consistent with other studies.^37^

While participants reported barriers and facilitators to participating in the trial, (parking, health literacy level of materials, and ease of undertaking home exercises and scheduling sessions) these are all well discussed in the wider literature. Transport and commuting is known to be a barrier for patients to access to healthcare.^38^ Health literacy of an individual can have a negative impact on health behaviour and clinical outcomes (e.g., poor self-management skills, non-adherence to treatment, increased healthcare costs).^39^ In our study, it highlighted the need to further simplify the instructions used in documents prepared for participants. Some participants thought home-based exercises were easy to perform, however others reported a lack of motivation to complete these at home. The complexity of exercises and patients’ motivations can impact on patients’ commitment to home-based exercise.^40^ Our findings suggest that by attending face-to-face sessions, some participants felt more motivated to perform the exercises at home. These findings suggest that a combination of supervised and home-based exercises is likely to be the most beneficial for improving patients’ long-term commitment to therapeutic exercises.

Clinicians’ perceptions about interventions tested within the trial also influenced patients’ perceptions about the efficacy of the prescribed exercises. Clinicians are likely to have a strong input into patients’ perceptions about their condition and the relevance of the treatment received.^41^ In this study, some clinicians were not completely comfortable with changing their practice to follow the protocol of the trial and the issues raised by participants are very similar to those raised in other literature.^11^ These findings highlight the need to have clinicians onboard with the planned intervention. Clinicians’ input at the design stage may help reduce perceived barriers and providing training and education in research and trial methods could reduce those barriers.^11^^,^^42^^,^^43^

Participants suggested presenting more information about the management of shoulder pain within the planned interventions, including the “dose” of home-based exercises. These suggestions will be incorporated by the research team when planning the full trial. Adding supporting material to participants could also address their requests for understanding why their shoulder hurts. Participants also showed interest in knowing the results of the feasibility trial. At the time of the interview, we had not completed the analyses of the trial, so we could not share findings with participants. We did, however, share the main findings with participants once data analyses and the report were completed. This is considered best practice in research and is an important aspect of dissemination of study results.^44^^,^^45^

This study has limitations. Nine participants opted to not take part in the study. It is possible that these participants had different perspectives and experiences about the feasibility trial. On the other hand, we reached data saturation during the interview of those 16 participants who agreed to take part in the study, so we consider it likely that we have identified the key issues faced by participants during the study. Further, we recorded limited information about participating interviewees demographics, so as to minimize burden, thus we cannot exclude the possibility of sociodemographic bias. Our findings suggest participants included in the study had heterogeneous occupations (which imply different levels of education and income). Despite the heterogeneous occupations held by participants, they all presented similar perspectives regarding the trial.

## Conclusion

This study explored participant perceptions of the feasibility trial which compared a tailored intervention to a standardised strengthening programme. Our findings suggest participants volunteered mainly to access healthcare for a specific issue that happened to match the trial requirements and to contribute to research. We found that participants valued the personalised care, perceived that their engagement within the trial improved their self-management and self-efficacy behaviour, valued the time spent with clinicians to receive interventions planned, as well as the empathetic environment of and education received during the trial. Participants recommended areas that could improve the design of the full trial including the health literacy level of written materials, making sure potential participants were aware of the time required to participate in a trial, and consideration be given to how to best encourage participant motivation to perform the exercises at home. We also found that clinicians’ perceptions of the trial influenced participants’ perceptions (in a negative way). Both the facilitators and barriers will require careful consideration in the future as the barriers may impact on reliability and validity of future trial results.

## Declaration of competing interest

None to declare.

## Appendix Supplementary materials

Image, application 1

## Footnotes

Supplementary material associated with this article can be found in the online version at doi:10.1016/j.bjpt.2024.101086.
