# Supplementary material for: Patients' perspectives on planned interventions tested in the Otago MASTER feasibility trial: an implementation-based process evaluation study
Source: Braz J Phys Ther. 2024 Jun 14;28(4):101086. doi: 10.1016/j.bjpt.2024.101086 (PMC11260924; doi:10.1016/j.bjpt.2024.101086)
Supplement: Supplementary file 1 [file mmc1.docx]

**Table S1. Interview guide.**

| Topic | Questions |
| --- | --- |
| Experiences with shoulder pain care | 1. What care have you received for your shoulder pain before you decided to take part in the study? 2. What were your expectations about the study? 3. Why did you decide to take part in the study? |
| Experiences throughout the study  and future clinical practice | 1. What is the added value of the care you received during the study compared to usual care (or care you have been receiving before the study)? 2. Were there barriers for taking part in the study? 3. How your expectations compared to the care you have received? 4. How does the care you have received during the study compare with the care you have received before participating in the study? 5. What, if any, drawbacks might there be? Did you feel like you had barriers to participate in the care during the study? |
| Future adoption of the care you have received during the study | 1. What, in your opinion, are the potential barriers patients may have to participate in the study / in the type of care you have received during the study? 2. Is there anything that you suggest could have made your rehab process easier or more manageable? 3. What, in your opinion, are the potential facilitators patients may have to participate in the study / in the type of care you have received during the study? 4. What resources would help you to enjoy/benefit more from the study? |
